# Client perspective assessment of women’s satisfaction towards labour and delivery care service in public health facilities at Arba Minch town and the surrounding district, Gamo Gofa zone, south Ethiopia

**DOI:** 10.1186/s12978-016-0125-0

**Published:** 2016-02-11

**Authors:** Zeritu Dewana, Teshale Fikadu, Abebe G/ Mariam, Misra Abdulahi

**Affiliations:** Arba Minch Health Science College, Arba Minch P.O.Box # 55, Arba Minch, Ethiopia; College of Public Health and Medical Sciences, Department of population and family health, Jimma University, Jimma P.O.Box # 230, Jimma, Ethiopia

**Keywords:** Delivery, Satisfaction, Arba Minch, Health service

## Abstract

**Background:**

A woman’s satisfaction with labour and delivery care service has a good effect on her health and subsequent utilization of the services. Thus knowledge about women’s satisfaction on labour and delivery care used to enhances the services utilization. The objective of this study was to assess the satisfaction of women’s towards labour and delivery care service and identify factors associated it at public health facilities in Arba Minch town and the surrounding district, Gamo Gofa zone, southern Ethiopia.

**Methods:**

Facility based cross sectional study was conducted among women who gave birth at public health facility. A total 256 women who gave birth during the study period were included in the study. Data was collected using a structured questionnaire. Satisfaction level was measured using a 5 point- likert scale questions. Data were entered using Epi data version 3.5.1 and analyzed using SPSS 20.0 statistical software. Factor analysis was employed for likert scale questions to extract factor represented each of the scale which facilitate treatment of variable as continuous for further analysis. Bi-variate and multivariable logistic regression analysis was employed to identify association between women’s satisfaction and predicator variables. Statistical significance was declared at P value <0.05 on final model. The strength of association was interpreted using the adjusted odds ratio and 95 % CI.

**Result:**

This study revealed that 90.2 % of women who gave birth in public health facilities were satisfied with labour and delivery care. Factors associated with women’s satisfaction with labour and delivery care services include: not attending formal education [AOR = 8.00, 95 % CI = (1.52, 12.27)] attending antenatal care four times and more [AOR = 5.00, 95 % CI = (1.76, 14.20)] waiting below 15 minutes to be seen by health professional [AOR = 3.37, 95 % CI = (1.14, 9.97)] and not paying for drugs and supplies [AOR = 6.19, 95 % CI = (1.34, 18.59)].

**Conclusion:**

Although majority of women were satisfied with the labour and delivery service they got, their level of satisfaction was influenced by educational status, number of ANC visits, waiting time, and payment for drug and supplies. Thus, public health intervention working on improving delivery care should consider these factors.

## Background

The health of women and children remain to be the major challenges in the world, particularly in developing countries. Globally an estimated 287,000 maternal deaths occur annually, 99 % of this occurs in developing countries, particularly in Sub-Saharan Africa which accounted for 56 percent of the total deaths. Of these deaths most are caused by complications during or just after delivery and the vast majority of the complications are avoidable coordinated interventions [1–3].

Ethiopia is one of the countries that have the highest maternal mortality rates in the world which is estimated to be 676/100,000 live births, every 1000 live births about seven women die during pregnancy, childbirth, or within two months of birth [4].

Maternal morbidity and mortality highly decrease by safe delivery and skilled birth attendant at every birth thus women’s satisfaction with the service was crucial [5, 6]. Improving maternal health and decreasing maternal mortality fall into three prevention strategies among this strategies increasing emphasis on client satisfaction with care in order to improve clients’ adherence to the service and improving labour and delivery techniques [4, 7] despite this maternal morbidity and mortality did not decrease and coverage of skilled birth attendant during labour and delivery care did not increase as required.

Satisfaction influences clients’ use of health services and experience of clients in obtaining care is believed to be an important predictor of future use of services as well as to influence care-seeking behavior among others in the community [8–11]. Comprehensive knowledge about women’s satisfaction by labour and delivery care in local context is vital to increase delivery service utilization, to develop new intervention strategies and strengthen the existing intervention programs to reduce maternal morbidity and mortality.

Therefore the aim of this study was to assess women’s satisfaction towards labour and delivery car service and its associated factors at public health facilities in Arba Minch town and the surrounding district.

## Methods

### Study setting

The study was conducted in Arba Minch town and the surrounding district, Gamo Gofa zone, south Ethiopia. It is located 505 Km south of Addis Ababa (*the capital city of Ethiopia*). The town and the surrounding district had a total population of 300,057 in the year 2013/14 which is projected from 2007 Ethiopia Central Statistical Agency [12]. The area has one hospital and eight health centers providing an estimated 3876 labour and delivery care services annually free of charge.

### Study design

Facility- based cross sectional study was conducted from April 1 to 25, 2014.

### Sample size

Sample size was calculated using Epi info version 7 assuming the proportion of women who were satisfied with delivery care service to be 82.9 % taken from other study [13], confidence level of 95 %, 5 % degree of precision, and 10 % for non-responses rate. The calculated sample size was 229; however, all (256) women’s who gave birth at public health facilities during the study periods were included.

### Sampling procedure

The sample was allocated proportionally to all public health facilities based on monthly average number of women who gave birth in each facility for the year 2012/2013. All women who gave birth during the study period were interviewed by considering women who refused to participate in the study as non-respondent.

### Data collection instrument

Data collection instrument was a structured questionnaire. Labour and delivery care service satisfaction related questions were adopted from different literatures and presented using a 5- point likert scale (1-very dissatisfied, 2-dissatisfied, 3-neutral, 4-satisfied, and 5-very satisfied). The questionnaire was initially prepared in English and translated into local language, then translated back to English to check for consistency. The instrument was pre-tested in 5 % of sample size in adjacent district public health facility. The reliability of the questionnaire checked using Cronbach’s Alpha and reliability coefficient was 0.87.

### Data collection

Data were collected using structured questionnaire through face to face interview at exit with participant. The data were collected by nine data collectors who have diploma in midwifery after two days of training. Filled questionnaires were checked daily for its completeness by supervisors.

### Data analysis

Data were checked for completeness, edited, coded and entered into Epi data version 3.1 and exported to SPSS 20.0 statistical software for analysis. Factor analysis was employed for likert scale questions to extract factor represented each of the scale which facilitate treatment of variable as continuous for further analysis. Assumptions were checked: Kaiser-Meyer-Olkin (KMO) measure of sampling adequacy was >0.5 and bartlet test of significance was significant at p < 0.05. Eigen value >1 was taken to select components.

All variables were checked for their communality value that is proportion of the variance in the original variables that is accounted for by the factor solution at >0.5 and variables with <0.5 value were removed from analysis and the procedure was repeated again. However, in this study all communality variables fulfilled the selection criteria except one component which was excluded. Finally, variables were reduced to one component to measure client satisfaction towards delivery care service with Cronbach’s Alpha (reliability coefficient) value of greater than 0.7. For the overall satisfaction level, those who were satisfied in greater or equal to factor mean score of the items were categorized under “satisfied” and those who were satisfied in less than factor mean score of the items were categorized as “un satisfied”. Descriptive statistics was computed for the study variables and frequency distribution tables were used to describe most of the findings. Variables with p-value less than 0.25 in bivariate analysis were entered to multivariate logistic regression using backward stepwise methods to determine factors independently associated with women’s satisfaction. Odds ratios (95 % confidence intervals) were calculated to determine the association between women’s satisfaction and independent variables. Model fitness was assessed using Hosmer and Leme show test (p = 0.935). Results were presented using tables, figures and narratives.

### Ethical consideration

The ethical clearance was obtained from Jimma University, Ethical review board. Letter of cooperation to health facility was obtained from Gamo Gofa zone health office to town and the surrounding district health office. Then letter of cooperation to the respective hospital and health centers was written by town and the surrounding district. Informed (verbal) consent was obtained from the study participants. To maximize privacy of responses, staffs were not present during data collection.

## Result

A total of 256 women were participated in this study. More than three forth (77.7 %) of the women were found to be in the group 20 – 34 years with the mean age of 24 (SD ± 5.0). Nearly one third (32.8 %) of the respondents have attended grade 1-8 while (23.4 %) did not have formal education. Half (50.6 %) of the respondents were protestant by religion and majority (90.6 %) of them were married. More than half 145(55.9 %) of the participant were from urban. and 170(78.9 %) had plan to give birth at health facilities (Table 1).

**Table 1:** Socio demographic characteristic of the respondent, Arba Minch town and the surrounding district, Gamo Gofa zone, South Ethiopia, April, 2014

Variable	Number	Percent
Age of mother (in year)		
< 20	44	17.2
20 – 34	199	77.7
35 – 49	13	5.1
Educational status		
No formal education	60	23.4
1 – 8	84	32.8
9 – 12	38	14.8
College and university	74	28.9
Religion of mother		
Orthodox	117	45.7
Protestant	132	51.6
Muslim	7	2.7
Marital status of mother		
Single	24	9.4
Married	232	90.6
Place of resident		
Urban	143	55.9
Rural	113	44.1
Family size		
Below or equal to 5	201	78.5
Above 5	55	21.5
How did you come to this facility?		
Planned	170	78.9
Family/neighbor	32	12.5
Referred from other facility	54	21.1

### Obstetric history

More than half 144(56.3 %) of the respondent were attended the recommended antenatal care visits, 187(73 %) had under 5 children, 237(90.6 %) delivered by SVD, 147(57.4 %) stayed in labour for less than 12 hours and 245(95.7 %) delivered alive child.

Most of the mother 174(68.0 %) reported traveling less than one hour to get to health facility, 138(53.9 %) by public transport, l45(56.6 %) delivered in hospital & payed for drug and supply. Also majority 173(67.6 %) of the respondent received care by female health professionals as, by midwifes [183(71.1 %)], 196(76.6 %) waited less than or equal to 15 mints to be seen by health care provider (Table 2).

**Table 2:** Obstetric related characteristic of the respondent, Arba Minch town and the surrounding district, Gamo Gofa zone, South Ethiopia, April, 2014

Variable	Number	Percent
Parity		
One	124	48.4
Two to five	124	48.4
Above five	8	3.1
Number of Under 5 children		
One	187	73.0
Two	69	27.0
Last pregnancy was planned		
Yes	184	71.9
No	72	28.1
Ante natal care visit		
Less than four visit	112	43.8
Four and more	144	56.3
Duration of labor		
< 12 hour	147	57.4
12-24 hours	71	27.7
> 24 hours	38	14.8
Presence of support persons during labour		
Yes	130	50.8
No	126	49.2
Type of delivery		
SVD	232	90.6
C/S	24	9.4
Newborn out come		
Alive	245	95.7
Dead	11	4.3
Facility distance by waking		
One hour or less	174	68.0
One to five hours	67	26.2
Above five	15	5.9
Means of transportation		
Ambulance	58	22.7
Waking	60	23.4
Public transportation	138	53.9
Waiting time to see by provider		
With in 15 minute	196	76.6
More than 15 minute	60	23.4
Payment for drug and supply		
Yes	145	56.6
No	111	43.4
Care provider sex		
Female	173	67.6
Male	83	32.4
Place of delivery		
Hospital	145	56.6
Health center	111	43.4
Care provider qualification		
Doctors	42	16.4
Midwifery	183	71.1
Nurse	20	7.8
Other	11	4.3

### Factors associated with women’s satisfaction with delivery service

Overall women’s satisfaction in this study was 90.2 % with factors mean index score of -1.67 (SD ± 1). respondents who did not attending formal education were 8 times more likely satisfied than those attending college and university education [AOR = 8.00, 95 % CI = (1.52, 12.27)]. Respondents who had four and more antenatal visits were 5 times more likely to be satisfied than their counterparts [AOR = 5.00, 95 % CI = (1.76, 14.20)]. Respondents who waited below 15 minutes to be examine by health professional were 3 times more likely satisfied than those who waited more than 15 minutes [AOR = 3.37, 95 % CI = (1.14, 9.97)]. Respondents who did not pay for drugs and supplies were 6 times more likely to be satisfied compared to those paid (Table 3).

**Table 3:** Factors independently associated with client satisfaction towards delivery care service at public health facility in Arba Minch town and surrounding district, Gamo Gofa zone, South Ethiopia, April, 2014

Variable	Satisfied No(%)	Unsatisfied No(%)	COR(95 % CI)	AOR(95 % CI)
Educational status				
No formal education	58(96.70)	2(3.30)	6.77(1.47,31.09)^a^	8.00(1.51,12.27)^a^
1-8	81(96.40)	3(3.60)	6.30(1.73,22.91)	4.36(0.99,19.28)
9-12	32(84.20)	6(15.80)	0.68(0.44,3.55)	1.071(0.31,3.76)
College & university	47(23.70)	27(46.60)	1	1
ANC visit				
Less than four	95(84.80)	17(15.20)	1	1
Four and more	136(94.40)	8(5.60)	3.04(1.26,7.34)^a^	5.00(1.76,14.20)^a^
Waiting time				
Within 15 minit	181(93.30)	15(7.70)	2.41(1.01,5.69)^a^	3.37(1.14,9.97)^a^
> 15 mint	50(83.30)	10(16.70)	1	1
Payment status				
Yes	123(84.80)	22(15.20)	1	1
No	108(97.30)	3(2.70)	6.44(1.88, 12.11)^a^	6.19(1.34,18.59)^a^

^a^Significant, 1 reference category

## Discussion

In this study 90.2 % of the respondents were satisfied towards labour and delivery care service provided. This finding is higher when compared to other studies (82.9 %, 61.9 %) conducted in different parts of Ethiopia [13, 14]. This difference could be due to study setting difference, more availability of health service facilities and could also be the methodological difference in which we used factor analysis to set the cutoff point for satisfaction.

Participants not attending formal educational were more likely to be satisfied than mothers attending college and university, it is true that when educational level increase awareness about delivery care increase so their expectation also become high & not satisfied with minimum compromise to the care. This is in line with the study finding reported from Southern Ethiopia and Amhara region [13, 14] but education was not associated with client satisfaction on a study conducted in Kenya [15] this difference could be probably due to study setting in which their conduct in informal settlements.

In this study those participants attending the recommended ANC visit were more likely to be satisfied than attending less than the recommended, but ANC visit had no any association with satisfaction by the studies done in different part of Ethiopia [13, 14]. This difference could be probably due to ANC visit classification difference in this study ANC visit classified as less than four visits and four and more visits but other studies classified as attended and not attended. The level of satisfaction is also related to the payment status, nonpaying clients were more likely to be satisfied than paying clients. This is in line with a study conducted in Amhara region [14]. This may be related to the fact that their expectation free of charge for labour and delivery car at public health facilities decrease worries about their economic status difference which may increase the satisfaction level.

Association with waiting time to be seen by health care provider were those wait less than 15 minutes were more satisfied than those waiting for longer time which is consistent with studies conducted in Ethiopia, Uganda & Bangladesh [8, 14, 16] which is encouraging.

## Conclusion

In this study educational status, number of antenatal care visits, waiting time to be examined by health providers and payment for drugs and supplies for labour and delivery care were independently associated with level of mother’s satisfactions towards delivery care. Thus, intensifying intervention programmer and stakeholder working on labour and delivery care practice should focus on these determinants to reduce maternal mortality and morbidity and realize millennium development goals.
